# The Positive Legacy of Vision Loss: Pathways to Posttraumatic Growth

**DOI:** 10.1093/geroni/igaa057.2094

**Published:** 2020-12-16

**Authors:** Corinna Tanner, Michael Caserta, Jia-Wen Guo, Margaret Clayton, Paul Bernstein, Julia Kleinschmidt

**Affiliations:** 1 Brigham Young University, Provo, Utah, United States; 2 University of Utah, Salt Lake City, Utah, United States; 3 University of utah, Salt Lake City, Utah, United States

## Abstract

This mixed method study describes posttraumatic growth (PTG) accruing form experience with vision loss caused by severe age related macular degeneration (AMD) and explores relationships between depression, social support, and cognitive processing, on the path to PTG. Research describing the psychological and social issues surrounding AMD has focused on negative outcomes. However, learning from highly challenging experiences, such as vision loss, can offer benefits. In this study, these included an increased sense of personal strength, increased spirituality, and empathy for others (all domains of PTG). 89 participants with severe vision loss (mean age = 85.3 years, age range = 74–98 years) completed the interviewer-administered composite questionnaire, which identified elements of Tedeschi and Calhoun’s model of PTG. Relationships between variables were examined using path analysis. Findings were contextualized with data from 15 qualitative interviews. Findings underscored the importance of supportive others and deliberate cognitive processing in the path to PTG.

